# Supplementary material for: Feasibility of management of hemodynamically stable patients with acute myocardial infarction following primary percutaneous coronary intervention in the general ward settings
Source: PLoS One. 2020 Oct 9;15(10):e0240364. doi: 10.1371/journal.pone.0240364 (PMC7546471; doi:10.1371/journal.pone.0240364)
Supplement: S1 Table — BMI, body mass index; CCU, coronary care unit; CI, confidence interval; HR, hazard ratio; MI, myocardial infarction. Age, gender, Ambulance use, Killip classification, Anterior MI and using CCU on admission were entered into a multivariable model. (DOCX) [file pone.0240364.s001.docx]

**Table S1. Multivariable analysis for factors associated with 30-day in-hospital mortality**

|  | Multivariable | |
| --- | --- | --- |
| Variables | HR (95% CI) | *P* value |
| Use of CCU on admission | 1.12 (0.66 - 1.91) | 0.672 |
| Age (years) | 1.06 (1.03 - 1.09) | <0.0001 |
| BMI (kg/m^2^) |  |  |
| <18.5 | 1.03 (0.49 - 2.15) | 0.941 |
| 18.5-25.0 | ref |  |
| >25.0 | 0.63 (0.33 - 1.20) | 0.160 |
| Female | 1.56 (0.93 - 2.61) | 0.089 |
| Ambulance use | 1.71 (0.89 - 3.27) | 0.108 |
| Source of admission |  |  |
| Home | ref |  |
| Another hospital | 1.52 (0.60 - 3.84) | 0.374 |
| Nursing home | 4.11 (1.93 - 8.72) | 0.0002 |
| Killip classification |  |  |
| 1 | ref |  |
| 2 | 1.13 (0.62 - 2.04) | 0.692 |
| 3 | 2.29 (1.19 - 4.43) | 0.014 |
| Unclassified | 2.91 (1.22 - 6.94) | 0.016 |
| Anterior MI | 1.19 (0.74 - 1.94) | 0.473 |

BMI, body mass index; CCU, coronary care unit; CI, confidence interval; HR, hazard ratio; MI, myocardial infarction.
