# Supplementary material for: Feasibility of management of hemodynamically stable patients with acute myocardial infarction following primary percutaneous coronary intervention in the general ward settings
Source: PLoS One. 2020 Oct 9;15(10):e0240364. doi: 10.1371/journal.pone.0240364 (PMC7546471; doi:10.1371/journal.pone.0240364)
Supplement: Attachment — Submitted filename: Response to reviewer_0923.docx [file pone.0240364.s003.docx]

**Responses to the comments of Reviewer #1**

Thank you for the careful and comprehensive review of our manuscript. In response to the Reviewer’s comments and recommendations, we have answered the questions in a point-by-point fashion and revised our manuscript as follows. Changes we made are written in red in the manuscript.

**1. What is the accuracy of diagnostic codes form STEMI in your dataset?**

**2. Where there differences between STEMI vs NSTEMI pts?**

> Because of the lack of information in the DPC database, it is impossible to identify patients with STEMI/NSTEMI. We acknowledge that this is the one of major limitations of our study. Thus, we have added the following sentence in the Limitation section.

Page 12, Line 22-23

“In the present study, acute MI includes STEMI and NSTEMI, both of which could not be distinguished.”

**3. In the STEMI cohort, were there difference by delays to revascularization, primary PCI vs lytics, or success of reperfusion?**

> In-hospital mortality in patients with STEMI depends on initial strategies (i.e. timely primary PCI, late primary PCI, or a pharmaco-invasive strategy) as shown in the FAST-MI program [Eur Heart J. 2020;41:858-66]. We believe that this is of clinical interest. However, because of the lack information in the DPC database, STEMI patients could not be identified in the present study. Thus, we have amended the following sentence in the Limitation section.

Page 12, Line 18-21

“First, this was a retrospective study using the DPC administrative database, which does not provide detailed clinical information including past medical history, laboratory findings, reperfusion strategies, and ST-segment elevation on electrocardiogram.”

**4. Interhospital transfers may represent a sicker cohort of patients. How were these patients handed in the analysis?**

> According to the reviewer’s suggestion, we have added the data into Table 1 and 2. When simply dividing patients into two groups (admission from home vs. transfer from another hospital), there was no significant difference in 30-day mortality (1.7% vs. 2.5%, p=0.325). However, we found that patients admitted from nursing home had significantly higher mortality. Therefore, we divided patients into three groups (admission from home vs. transfer from another hospital vs. admission from nursing home), resulting in significant difference in 30-day mortality (1.6% vs. 2.5% vs. 16.4%, p<0.001). Accordingly, we have included the source of admission as a confounding factor into Cox proportional hazard model and re-conducted PSM analysis. Nevertheless, the basic results did not change. We have amended the Methods and Results sections, and Table 1, 2, S1, Figure 1 and 2.

Page 2, Line17-18

“hazard ratio: 1.12, 95% confidence interval 0.66 - 1.91, p=0.67”

Page 6, Line 7-13

“We included the following variables as confounding factors: age, sex, ambulance use, source of admission (home, another hospital, and nursing home), body mass index, Killip classification on admission, the presence of anterior MI, and comorbidities (hypertension, dyslipidemia, diabetes mellitus, and chronic kidney disease).”

Page 7, Line 10-11

“source of admission”

Page 7, Line 16

“transfer from another hospital”

Page 8, Line 7

“after: 1.8% vs. 1.1%, p=0.08”

Page 9, Line 8-9

“HR 1.12, 95% CI 0.66-1.91, p=0.67”

Page 9, Line 9

“admission from nursing home”

Page 9, Line 12

“PSM analysis: log rank p=0.17; Cox proportional-hazards regression analysis: HR 1.02, 95% CI 0.63-1.69, p=0.91”

Page 10, Line 7

“11 [9-15] vs. 12 [9-16] days, p<0.001”

Page 10, Line 8-9

“1780000 [1480000-2150000] vs. 1830000 [1560000-2240000] JPY, p<0.001”

Page 11, Line 5-6

“1.8% vs. 1.1%”

**5. Do the authors have any information on patients requiring intravenous vasoactive agents or ventilation?**

We appreciate the reviewer’s meaningful comment. We have added the information of intravenous vasoactive agent (vasopressor and vasodilator agents) in Table 2. Also, we have added the data regarding ventilation in Table 2. There was no significant difference in the use of ventilator between the GW and CCU groups. On the other hand, the GW group had higher incidence of use of intravenous vasopressor and vasodilator agents than CCU group before and after PSM. We have amended the Methods section accordingly.

Page 6, Line10-13

“The use of an intra-aortic balloon pump and extracorporeal membrane oxygenation beyond the day of admission and ventilator, intravenous vasopressor agents (catecholamine, etc.) and intravenous vasodilators (nitrates, carperitide, etc.) were selected as surrogates of disease severity.”

**6. What were the potential cost savings if this was applied more broadly?**

In this present study, 5736 out of 19426 patients (29.5%) with acute MI were hemodynamically stable, and 3488 of 5736 patients (60.8%) were admitted to CCU rather than GW. Total hospitalization cost was 80000JPY higher in the CCU group than in the GW group in this study. Considering the number of patients diagnosed as acute MI each year in Japan (n=82000), if all hemodynamically stable patients with acute MI defined in the present study were initially managed in GW, 1176560000JPY (approximately 11 million US dollars) might be saved in Japan each year, although this is a very rough estimate and may be beyond the scope of the present study. We have added the following sentences in the Discussion section.

Page12, Line9-12

“In addition, it is conceivable that management of hemodynamically stable patients with acute MI in GW offers opportunities for other patients who require intensive care in CCU. Considering the limited capacity of ICU beds, this would be beneficial to improve health resource allocation.”
